# Cellular Landscapes in Watercolor

**DOI:** 10.5210/jbc.v40i1.6627

**Published:** 2016-02-26

**Authors:** David S. Goodsell

**Affiliations:** 1 The Scripps Research Institute

## Abstract

The molecular structure of cells is currently not accessible to most experimental techniques, but a view may be synthesized from data on molecular structure and cellular ultrastructure. This article describes the preparation and process for creation of a painting that shows the molecular environment of a portion of a living cell.

## Introduction

**Figure 1:**
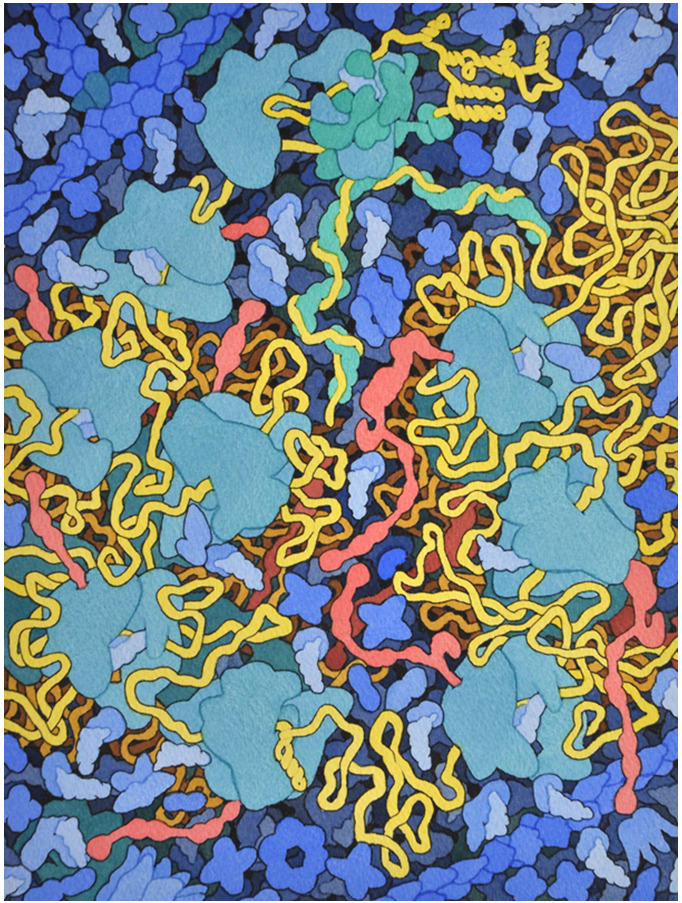
HIV Translation and Frameshifting

Fifteen years ago, I had the pleasure of writing a short article for the *Journal of Biocommunication* describing some of the techniques I used for the molecular and cellular illustrations in the book, "The Machinery of Life" (Goodsell 2000; Goodsell 2009). These illustrations simulate a view of the cellular mesoscale, taking a portion of a cell and depicting its molecular composition (see, for instance, (Goodsell 1991; Goodsell 2009)). At the time, most of the illustrations, including those in the book, were in black-and-white, since color was often prohibitively expensive in scientific trade publications. For the second edition of the work, as well as a number of educational settings, I have subsequently developed a color approach to these cellular landscapes, which allows far better comprehension and depiction of more complex scenes. In this paper, I will present some of the preparation and process for creating these cellular landscapes in watercolor, using a recent painting of protein synthesis in an HIV-infected cell (Figure 1 ).

## Methods

### Gathering Supporting Information

I try to make these paintings as accurate as possible, so typically half the time for creating a painting is devoted to gathering information to support the structures of the molecules, their interactions, and how they are arranged in the ultrastructure of the cell. This stage often has the feel of a treasure hunt. There are a few sources of information that provide consistent results, but often the details of a particular system must be gathered from many scattered sources in the primary literature.

The painting shows an entire HIV polysome in the process of being translated into viral proteins. The HIV RNA is shown in yellow, and the growing viral proteins are in red. The initiation complex is at the top, and the RNA loops around, forming a circle with the poly-A tail bound to the initiation complex. The ribosome at bottom center is stalled by a short hairpin loop in the RNA, which causes it to fall off 90% of the time. In the other cases, the ribosome slips to a new reading frame and continues on the right-hand side of the picture to build a longer viral protein.

**Figure 2:**
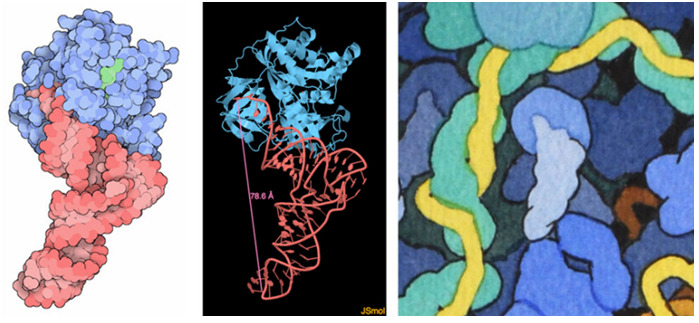
Determining Molecular Shape and Size.

The RCSB Protein Data Bank (PDB) and UniProt are my first stops for structures. The PDB has atomic structures for tens of thousands of biological molecules, and many tools for exploring them (http://www.rcsb.org ) (Berman 2000). When I began doing these paintings, I created an archive of pictures drawn at a consistent scale, to use as resources for depicting each molecule, such as the transfer RNA complex shown here (Figure 2 , left). One key aspect of rendering these images is to use orthographic projection, so that perspective effects do not distort the size and shape. Most currently available molecular rendering tools use perspective in their default views. As part of this work, I created a poster with the PDB, showing many common molecules drawn at a consistent scale. This has been recently updated and is available at:

http://www.pdb.org/pdb/education_discussion/educational_resources/mol-mach-2014-poster.pdf . More recently, I often display a particular molecule using the interactive program JSmol (http://jsmol.sourceforge.net ), which is available for each entry at the RCSB PDB website, and I measure a few distances to get the size right (Figure 2 , center). This provides enough information to create the simplified outline for the painting (Figure 2 , right).

A digital image of an atomic structure is shown at left, rendered in orthographic projection at a defined magnification, provides size and shape information for an individual molecule (tRNA and elongation factor Tu, entry 1ttt at the Protein Data Bank (Nissen 1995)). Interactive visualization tools such as JSmol, shown in the center, allow rapid viewing and measurement to define molecular size and shape. The tRNA and elongation factor in the painting are shown on the right.

UniProt (http://www.uniprot.org ) is another essential tool in this search for information (UniProtConsortium 2014). Many proteins are flexible and are composed of multiple connected parts, and often the PDB includes only the portions of these molecules that are amenable to structural analysis. UniProt provides an easy way to look up a particular protein by name, and see its overall molecular weight, predicted and observed domain structure, annotations about interactions with other proteins, and links to experimental and modeled atomic structures, if available. For instance, for this painting, I looked up the viral gag-pol sequence to define the ordering of domains within the viral polyprotein (UniProt entry P04585).

For information related to molecular interactions, large protein complexes, and cellular ultrastructure, I rely heavily on PubMed (http://www.ncbi.nlm.nih.gov/pubmed ) to find research papers that describe the topics. I have found no short cut for this work--it requires the time to delve into the literature and try to become a temporary expert on the subject. This typically requires a mix of review articles to define all of the processes and molecules that need to be shown in a particular scene, and technical papers to chase down the individual players. In many cases, of course, information is still not available, and various levels of artistic license must be employed (Goodsell 2007). Typically, a very heterogeneous mix of papers is needed to support the painting. For instance, for this painting I found an electron microscope study of circular polysomes (Yazaki 2000), a 3D electron tomograph reconstruction of the ribosomal initiation complex (Sonenberg 2009), and modeled structures of the RNA hairpin involved in frameshifting (Brierley 2006). Available literature and diagrams from standard texts, endoscopic pictures from relevant publications, rectangular jar of transparent perspex /acrylic, thermofoam / thermocol pieces, colored transparent thick polythene / glass paper sheets, scissors, sharp razors, iron wires, pins, adhesive gum, etc.

### Designing and Creating the Sketch

**Figure 3:**
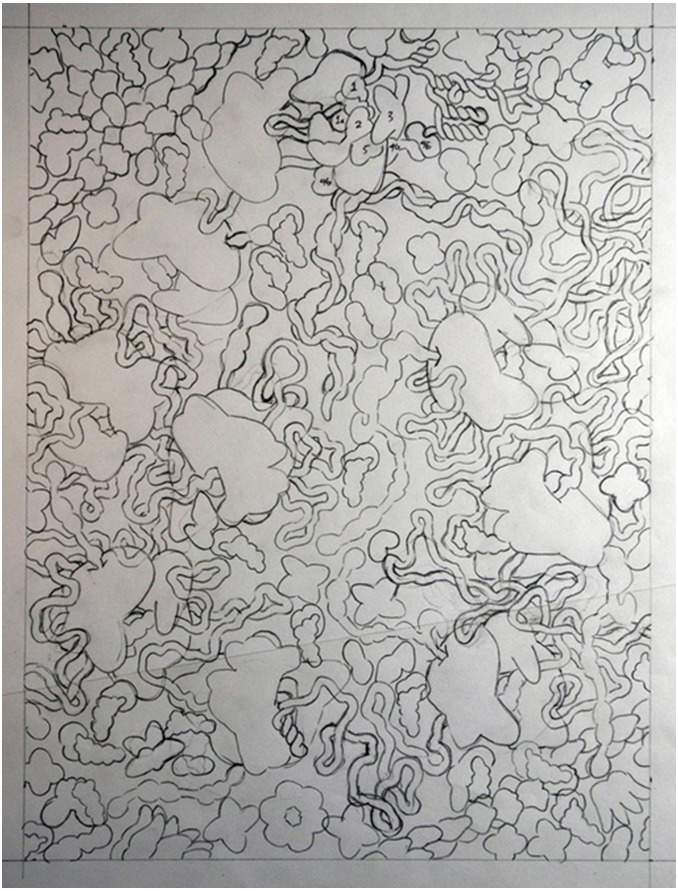
Sketch for "HIV Translation and Frameshifting"

The next task is to synthesize this diverse information into a sketch for the painting (Figure 3 ). I try to capture an important moment in the process being depicted. For instance, in this case, I wanted to show an entire polysome caught in the middle of the process of being translated, including the initiation of the process and the frameshifting that is important for the viral lifecycle. For clarity, the paintings typically include all of the macromolecules in the scene (proteins, ribosomes, RNA and DNA, etc.), and omit small molecules and water.

I typically work from ultrastructure to molecular structure. I define the largest players first, such as membrane-bounded compartments. In this case, I defined a circle of ribosomes, with the initiation complex at the top and a ribosome paused at the frameshift site at the bottom. After these were added, the viral RNA was woven between the ribosomes. Finally, all of the smaller soluble components (tRNA, protein synthesis factors, enzymes, etc.) were dotted into the remaining space, building up to the proper overall concentration.Finally, I transfer the sketch to Arches 300-pound watercolor paper. This beautiful paper is so heavy, and my washes of color are typically so small, that I don't bother with traditional stretching. I just tape it to a piece of foam core and I'm ready to go. For the transfer, I use an old mylar-backed carbon paper that was given to me by my grandfather when I was a child. I don't know what I'll do when my supply of this is exhausted--I can't imagine that it's still available.

### Watercolor Rendering

Many years ago, I exhibited one of my first paintings at a The Guild of Natural Science Illustrators (GNSI) show. One of the attendees approached me and asked: "Once you draw the outlines, then it's all just paint-by-number?" He was almost correct--in fact, the style is very simple: the colors are painted in, light to dark, and then the outlines are added at the end. I have chosen this simple rendering style for several reasons. When I was experimenting with the first of these paintings, I tried several highly rendered approaches, with shading and shadowing on each molecule. This yielded a very complex image with a lot of visual distractions. I find that the cartoony, flat-color approach that I use now makes it easier to comprehend the whole scene. Equally as important, this rendering style is quite fast to paint, so I'm able to do paintings with high levels of complexity. Finally, I want to make a tight connection with the style that I use for the digital renderings of individual molecules (as in Figure 2 ), since I often present the paintings along with images of the component molecules.During a question and answer period at a recent Biocommunication Academic Meeting (BAM) meeting in Toronto, my approach to color was described as an "intuitive palette." I typically use blues and greens for the cytoplasm and membranes, so they tend to be the predominant themes, built from cobalt blue and ultramarine, viridian, and cadmium yellow. I often use yellows and reds to highlight important molecules, using cadmium yellow and winsor red. For instance, in this series of illustrations I used yellow for the viral RNA and red and magenta for viral proteins. Extracellular molecules are often in earth tones, with a mix of yellow ochre and burnt sienna. Vandyke brown is used to create the dark tones for background molecules.

**Figure 4:**
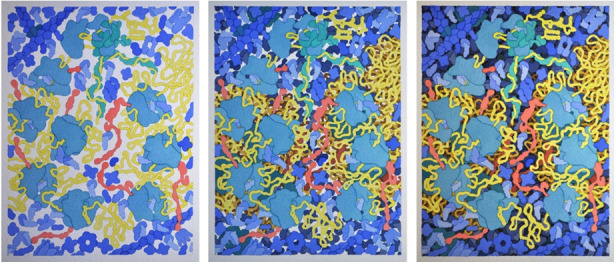
Three States of the Painting.

Color is added with a uniform wash for each molecule. The foreground, as defined by the sketch, is added first (left). Then a darker layer of molecules is added extemporaneously behind that (center), and a third, even darker layer fills the remaining visible space between molecules (right). Because of the transparent nature of watercolor, the color values are difficult to judge until the very last step, and may require some tuning at the end.

### Finishing Touches

The final step in these illustrations is to add outlines with a technical pen (typically size 00). In keeping with the overall style, the outlines are very simple, with small gestural elements along the edges that hint at the subunit composition of complexes. In large protein complexes, such as the initiation complex at the top of this illustration, I often paint each subunit with a separate wash of color. The overlap between these washes creates a softer outline between the subunits, which I do not harden with an ink outline. I usually leave these soft outlines between subunits that are associated with one another, and use ink outlines to separate molecules that are not interacting.

For publication, I photograph the paintings using a digital camera, and clean the image up in Adobe Photoshop (Adobe Systems, Inc., San Jose, CA). I usually paint at twice the size that will ultimately be published, creating the painting at 2,000,000 X magnification, for a final published magnification of 1,000,000 X. This magnification is sufficient to see the shape of individual molecules, while allowing the depiction of enough of the cell to show the functional ultrastructure.

### The Move to Mesoscale 3D Modeling

**Figure 5:**
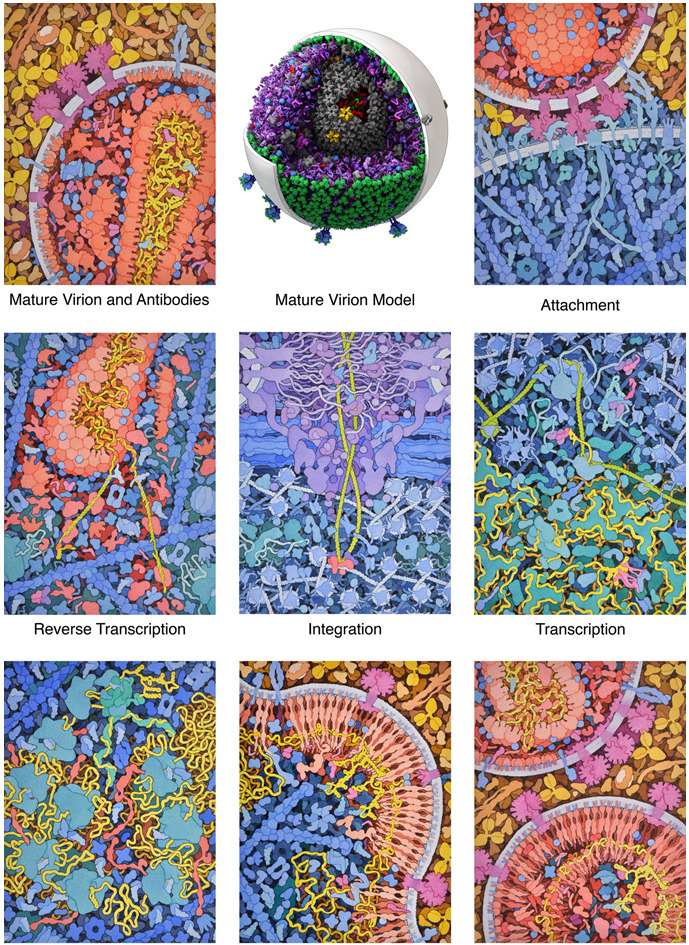
HIV Life Cycle.

As I write this manuscript, I am happy to report that the need for this type of hand-painted rendering is rapidly becoming obsolete. As part of the book project that included the painting shown here (Figure 5 ), Graham Johnson created a 3D model and rendering of the mature HIV virion, based on a model from the cellPACK software (Johnson 2014, Johnson 2014). This type of mesoscale modeling is becoming possible for increasingly complex cellular assemblies, combining experimental information on molecular structure, concentration, and distribution with ultrastructural information, to simulate a representative scene (or scenes). Looking at the figures for the book

shows some of the challenges that remain, that are relatively simple in hand-drawn illustration but still quite difficult to model in 3D, such as the complex infrastructure of the cytoskeleton and its connection to the plasma membrane, the mix of order and disorder in the nuclear pore, and the twists and turns of DNA packaging and transcription in the nucleus.

I created paintings of each step in the HIV life cycle to open chapters in a book on HIV drug targets, all created at a consistent style and magnification. They show some of the complexity that is inherent in mesoscale cellular subjects. The model at upper center, created by Graham Johnson, shows the current state-of-the-art for 3D mesoscale modeling.
